# Studies of the Behavioral Sequences: The Neuroethological Morphology Concept Crossing Ethology and Functional Morphology

**DOI:** 10.3390/ani12111336

**Published:** 2022-05-24

**Authors:** Vincent L. Bels, Jean-Pierre Pallandre, Eric Pelle, Florence Kirchhoff

**Affiliations:** 1 Institut de Systématique Evolution Biodiversité (ISYEB), Muséum National d’Histoire Naturelle, CNRS, Sorbonne Université, EPHE, UA, CP, 57 Rue Cuvier, 75005 Paris, France; jean-pierre.pallandre1@mnhn.fr (J.-P.P.); florence.kirchhoff1@ac-rennes.fr (F.K.); 2 Muséum National d’Histoire Naturelle, Direction des Collections, SPOT-Plateforme de Préparation Ostéologique, CP 55 Site Anatomie Comparée, 55 Rue Buffon, 75005 Paris, France; eric.pelle@mnhn.fr

**Keywords:** ethology, functional morphology, behavior, Tinbergen’s questions, feeding, ritualization, throat display

## Abstract

**Simple Summary:**

Behavioral sequences analysis is a relevant method for quantifying the behavioral repertoire of animals to respond to the classical Tinbergen’s four questions. Research in ethology and functional morphology intercepts at the level of analysis of behaviors through the recording and interpretation of data from of movement sequence studies with various types of imaging and sensor systems. We propose the concept of Neuroethological morphology to build a holistic framework for understanding animal behavior. This concept integrates ethology (including behavioral ecology and neuroethology) with functional morphology (including biomechanics and physics) to provide a heuristic approach in behavioral biology.

**Abstract:**

Postures and movements have been one of the major modes of human expression for understanding and depicting organisms in their environment. In ethology, behavioral sequence analysis is a relevant method to describe animal behavior and to answer Tinbergen’s four questions testing the causes of development, mechanism, adaptation, and evolution of behaviors. In functional morphology (and in biomechanics), the analysis of behavioral sequences establishes the motor pattern and opens the discussion on the links between “form” and “function”. We propose here the concept of neuroethological morphology in order to build a holistic framework for understanding animal behavior. This concept integrates ethology with functional morphology, and physics. Over the past hundred years, parallel developments in both disciplines have been rooted in the study of the sequential organization of animal behavior. This concept allows for testing genetic, epigenetic, and evo-devo predictions of phenotypic traits between structures, performances, behavior, and fitness in response to environmental constraints. Based on a review of the literature, we illustrate this concept with two behavioral cases: (i) capture behavior in squamates, and (ii) the ritualistic throat display in lizards.

## 1. Introduction

**Figure 1:**
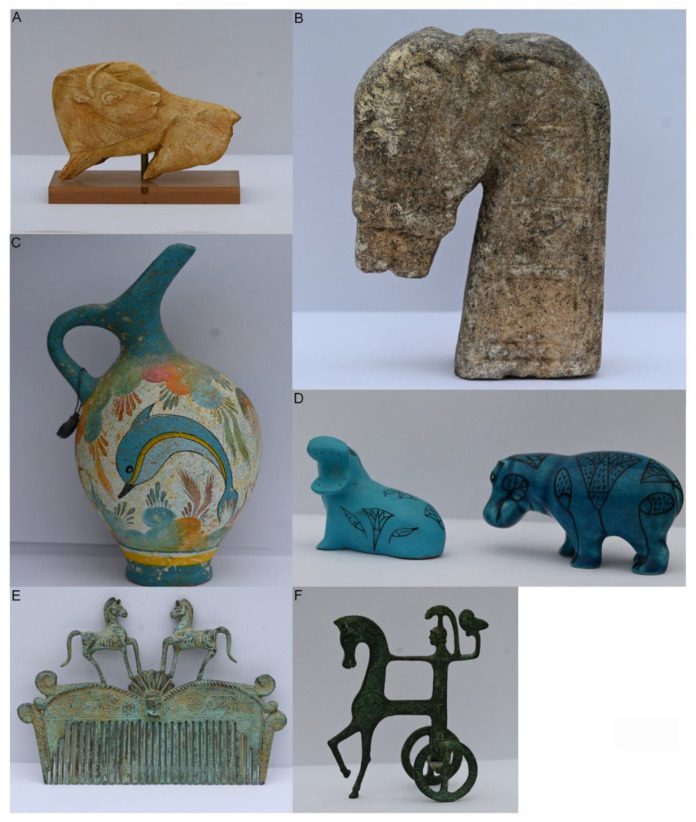
Represented postures of animal behavior on various substrates. (**A**) Magdalean bison. (**B**) Timor horse head. (**C**) Dolphin on a Minoan pottery. (**D**) Hippopotami from Ancient Egypt. (**E**) Horses on a Roman comb. (**F**) Chariot of the Goddess Athena from Ancient Greece.

Humans have formally developed representations of various postures and tentative movements to show the behaviors and/or animal expressions we can observe [1]. A variety of terrestrial and aquatic vertebrates illustrated on various substrates (e.g., stone, wood, ivory) have been represented since the earliest days in all human cultures. Probably, one of the first descriptions of animal movements was depicted in various parietal arts by representations of their behaviors (e.g., feeding and vigilance behaviors in large herbivorous mammals, predation behaviors in carnivores), and their “psycho-physiological” status through facial expressions (e.g., head expressions in hunting Felidae) [2,3]. For a long historical period, the representation of postures (Figure 1) or a series of postures remained the unique way to illustrate behaviors [4,5] related to human activities, such as hunting, agriculture, war, religious ceremonies, entertainment (e.g., feasts, circus arena), and torture. The illustrated postures of locomotion in many vertebrates (e.g., dogs, cats, horses, cows, elephants) are typical examples of “frozen” representations of behavioral sequences. All these representations are limited by human perceptual abilities (mainly visual) and/or knowledge of the animal’s behaviors (e.g., direct observations, memory, reports). The quantification of animal behaviors has been untimely supported by the technological rise of data recording, and analysis based on (i) photography and filming at different speeds initiated by Etienne Jules Marey (830–1904) and Eadweard Muybridge (1830–1904) [6,7,8,9]); (ii), embarked sensors [10,11,12,13,14]; (iii) imaging and computational technics [15,16]; and (iv) machine learning systems [17]. The investigation of animal movements recorded in behavioral sequences became the basis of our understanding of evolution and of the adaptation of the animal behavior to their environment [18]. Such investigation is the basis of the conceptual approach proposed in this paper.

## 2. Investigating Behavioral Sequences

**Figure 2:**
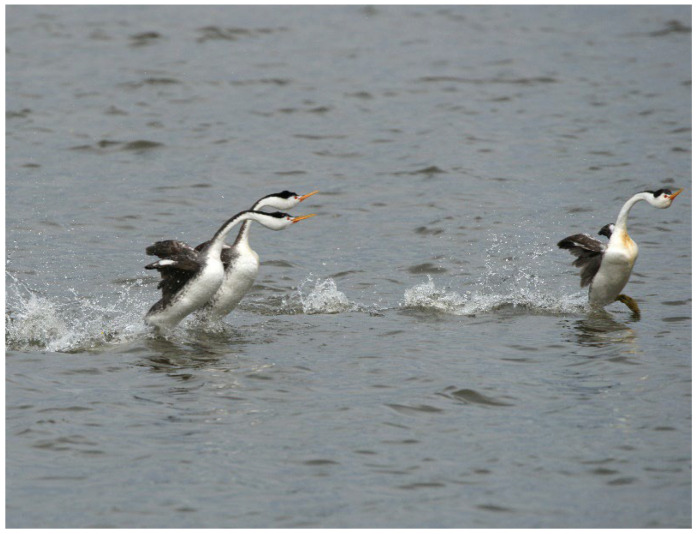
Courtship of *Aechmophorus clarki* showing that the water-running (rushing) behavior is performed by several animals together (photograph courtesy of A. Konter).

**Figure 3:**
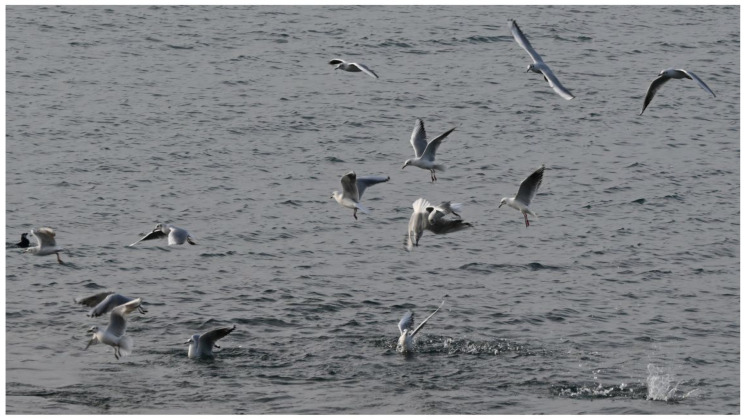
A group of seabirds preying on a fish shoal. All *Chroicocephalus ridibundus* plunge from different heights toward the fishes. Each bird plunges independently, and must avoid all others to catch its prey and get out of the water.

**Figure 4:**
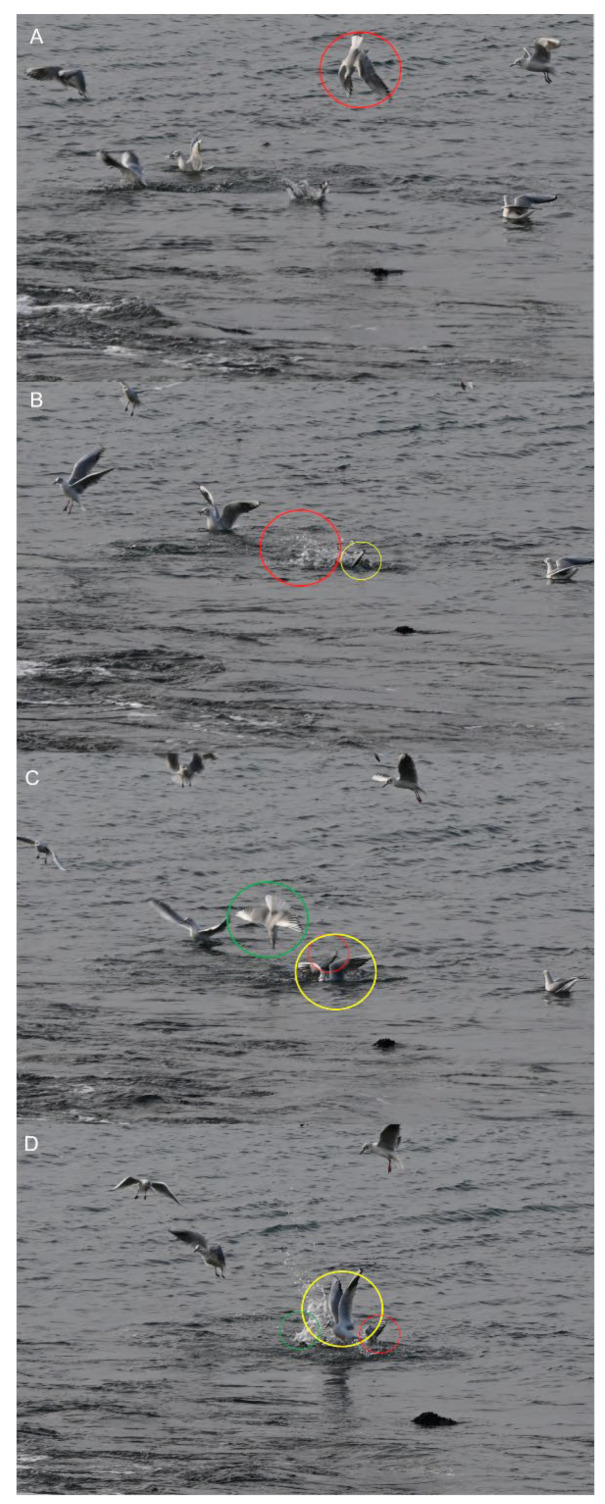
Motor sequence of three *Chroicocephalus ridibundus* closely preying on a fish shoal. (**A**,**B**) One individual plunges (red circle) toward the fish, while another begins to emerge (yellow circle). (**C**,**D**) An individual (green circle) plunges while another begins to emerge (red circle) and another (yellow circle) flies out of the water with the prey in its beak. Time between two frames: 0.01 s.

**Figure 5:**
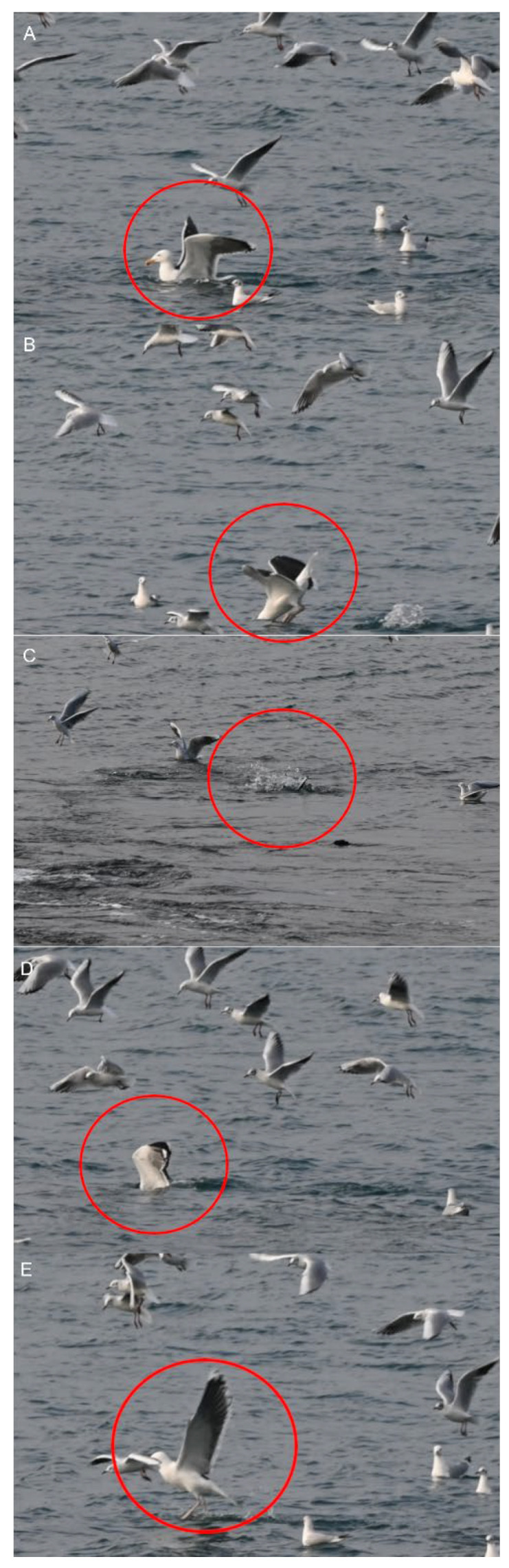
Motor sequence of *Larus marinus* (red circle) preying on the same fish shoal as *Chroicocephalus ridibundus* (see Figure 3 and Figure 4). (**A**,**B**) The swimming bird prepares to plunge by flapping its wings. (**C**,**D**) The bird plunges to catch the fish. The bird moves out of water (**E**)Time between two frames: 0.01 s.

Two types of behavioral sequences are investigated under experimental conditions and in free-living animals: (i) sequences of a single individual (e.g., locomotion, feeding), and (ii) sequences of a group of individuals (e.g., predation, schooling behavior, social cooperation and competition, migration, play). In the case of collective motions, the study of each animal sequence shows (i) the behavior of each individual, and (ii) the reciprocal effect of the action and postures of all the individuals [19,20,21,22,23]. In short, the data used to process collective motions are quantitative observations of the responses of a variable number of individuals [24,25]. This study permits the evaluation of when and how each individual synchronizes their gestures and increases the predictability of the incoming signal realized by each action of each individual on the others [26,27]. For example, the rushing motor action of one individual in the courtship of *Aechmophorus clarkii* reciprocally influences the response of the others [28,29] (Figure 2). In a group of sea birds preying on a fish shoal, the action of each individual is independent but can modify the strategy of the others (Figure 3). For instance, each *Chroicocephalus ridibundus* plunges independently toward the fish and then exits the water with the prey (Figure 4; Supplementary Material S1). Each animal must avoid all others. *Larus marinus* uses two strategies: (i) plunging from the air, and (ii) diving from swimming position (Figure 5). By using this last strategy, the bird probably has a better perception of the spatial position of most of the preying birds. Various individuals from the same species preying simultaneously on one food resource may show different “decision-making” variability, probably constrained by physical laws as suggested by the mechanoethology approach (e.g., sensation, limit, and energy [22]).

## 3. Behavioral Sequences in Ethology

**Figure 6:**
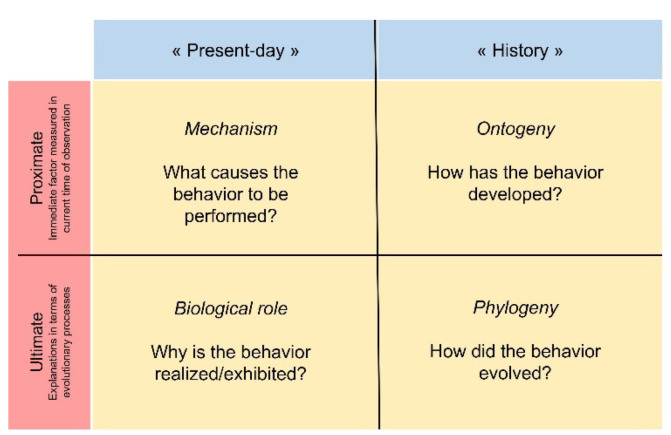
Classic schematic representation of Tinbergen’s four questions.

Behavioral sequences analysis is one (but not the only) relevant method to accurately quantify the repertoire of actions to answer Tinbergen’s four questions (Figure 6).

**Figure 7:**
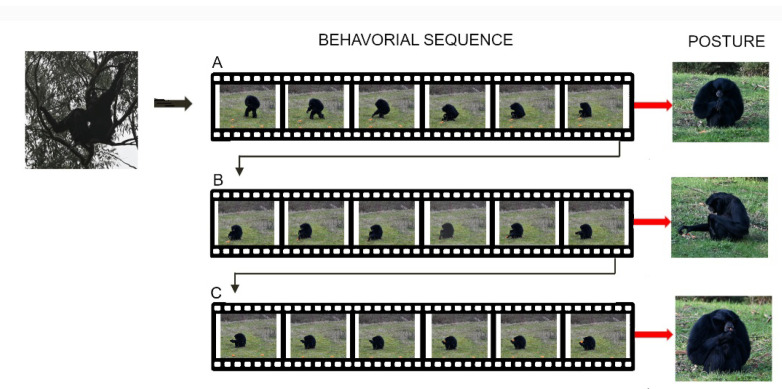
Sitting posture to reach the food (**A**), grasp it (**B**), and feed (**C**) in *Symphalangus syndactylus* result from the integration of combined motor actions (movements) of the axial, and appendicular systems (i.e., limb and hand) that are determined by analysis of animal sequences. The final posture of each behavior corresponds to the end of a sequence of movements of the whole body and/or one of its components under different motor controls.

Animal behaviors can be divided into movements, gestures, and postures that correspond to decision-making and result from motor actions [30,31]. Indeed, postures are the result of dynamic activities of the whole body or of one of its anatomical components [32]. For example, feeding is a complex behavioral sequence integrating cranial and post-cranial movements and postures [33], as demonstrated in primates (Figure 7).

**Figure 8:**
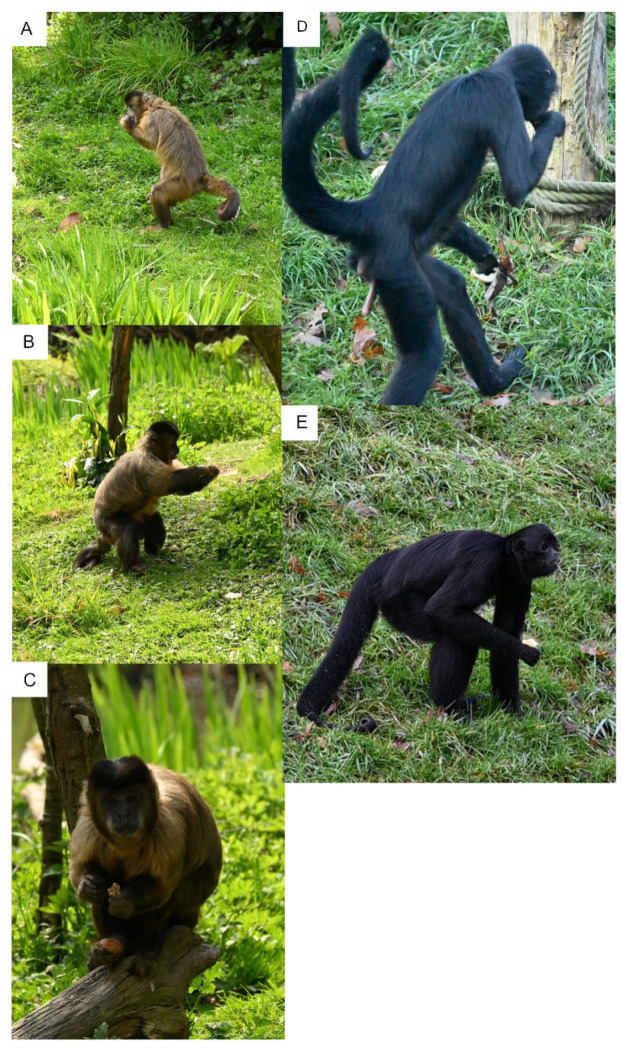
Various posture extracted at the end of body motor sequences during food transport and feeding by (**A**–**C**) *Cebus capucinus*, and (**D**,**E**) *Ateles fusciceps rubiventris.* (**A**) *C. capucinus* manipulating food and feeding in a bipedal posture. (**B**) *C. capucinus* manipulating food and feeding in a tripodal posture made by the two hindlimbs and the tail. (**C**) *C. capucinus* manipulating food and feeding in a “sitting” posture with food in the hindfoot (power grip). (**D**) *A. fusciceps rubiventris* feeding in a bipedal posture. (**E**) *A. fusciceps rubiventris* feeding in a tripodal posture made by the two hindlimbs and one forelimb. The structure of the limbs and the tail in both species influences the posture that the animals are able to use to handle food and feed.

**Figure 9:**
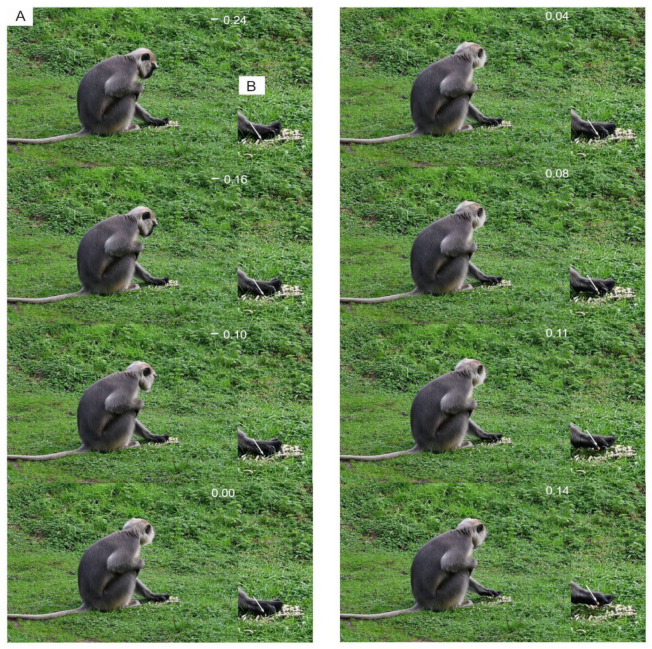
Typical non-rhythmic movement for single-handed food grasping in *Semnopithecus entellus.* (**A**) View the animal posture during grasping. (**B**) Close-up view of the hand showing the grasping movements of the digits. At time: −0.24 s, the animal examines the food source, and then scans the environment during the grasping sequence (Time: −0.16 to 0.14 s). The white arrow indicates the piece of food selected by the animal. Time 0.00 corresponds to the grasping of the food by the digits. The animal is filmed at 100 Hz.

Primates successively adopt a typical posture (e.g., sitting or tripodal posture, Figure 8), permitting them to freely extend the forelimbs to reach and grasp food, and to use their hands. Each movement involves the neuro-motor integration of different structures (e.g., axial and appendicular systems) with morphological properties (e.g., shape, length, articulation) under phylogenetic (historical) and ecological (e.g., degree of arboreality) constraints (Supplementary Materials S2 and S3). The hands are used to manipulate food in front of the trophic system, regardless of their morphological and mechanical traits, as in various tetrapods [34,35,36]. Each behavioral action resulting from the combined movements of different morphological systems (Figure 9) is also influenced by complex sensory filtered information from the food (e.g., food volume, size, texture [37,38,39,40,41]), the animal traits (e.g., age, sex, personality), and the environment.

Each entity (or unit) with a distinct function or a set of functions involved in the behavioral sequence was called “behavior-organ” by early ethologists [29]. Entities correspond to complex movements and gestures recorded through the lens of complementary disciplines (e.g., comparative and evolutionary ethology, phylogeny). Each of the behavioral entities is characterized by a neuro-motor pattern corresponding to precise activation and coordination of the muscular system at various scales (e.g., muscles, fascicles, or fibers) under the control of activated neural circuits called central pattern generators (CPGs). These entities are similarly identified in neuro-ethology and functional morphology by (i) the kinematic profiles (e.g., feeding) or particular representations such as gaits [42,43], and (ii) “motor-action” patterns (MP) controlled by hormonal and neuronal systems. Some entities are rhythmic as identified from several decades [44], and some others are not. Differentiating the types of entities is rather difficult in some behaviors. For example, food/prey catching is a particular “one-shot” pattern immediately followed by rhythmic manipulation and transport (Supplementary Material S4), and the hand is “frozen” in a typical posture at some point in the behavioral sequence for food grasping, while the fingers show rhythmic movement to screen the food resource (Supplementary Material S5).

## 4. Behavioral Sequences in Functional Morphology

**Figure 10:**
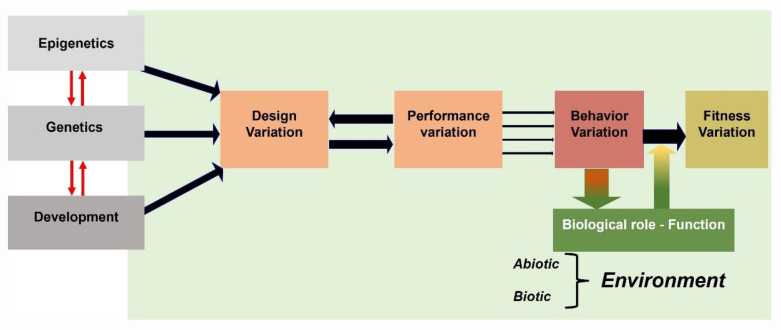
Illustration of an extended view of the Arnold’s paradigm [47] used in functional morphology, indicating the links between designs (structures), performances, behaviors, and fitness. Phenotypic traits of structures depend on complex interactions between processes relating genetics, epigenetics and development. A behavior is the result of movements of structures identified by a set of performances that reversely affect the properties of the design. Each behavior has a biological role in responding to properties of the environment [47].

In functional morphology (and biomechanics), the analysis of behavioral sequences makes it possible (i) to establish the properties (at various levels of its biological organization) of a phenotypic trait (e.g., structure), (ii) to determine the kinematic profiles and MPs governing the movements of this trait, and (iii) to discuss the links between the so-called “form” and “function” of this trait [45]. Such studies contribute to the understanding of evolutionary patterns and processes [46] in the approach that can be formalized in Arnold’s paradigm [47] linking structure, performance, behavior, and fitness (Figure 10) for an identified behavior (e.g., locomotion, feeding). This paradigm applies to all neuromotor patterns performed by animals, with the relationship between “behavior” and “performance” considered as distinct features [48] playing a key role in determining the behavior. Performance is extracted from precise studies of behavioral sequences investigated using various filming techniques (e.g., high-speed 16 mm and video films, high-speed X-ray films), embarked cameras, and diverse embarked sensors (e.g., electromyography, accelerometers and experimental studies [49,50,51]). Although largely discussed, fitness is simply defined here as the ability of the surviving organism to produce offspring that then (at least in part) enter the gene flow process in a population, etc. [48]. In this modified paradigm, “function” is represented as the biological role of the behavioral response to the environmental factors [52]. The functional characteristics measured at various biological levels (e.g., whole body, muscular and skeletal elements) of the design through its performances [52] permit hypothesis testing that, in short, allows us to respond to the Tinbergen’s questions (Figure 5). The behavior determined by designs (structures) movements quantified by these performances can also be determined as a “moving morphology” [53]. Actually, it is usual to measure these performances based on kinematic data (e.g., velocity, acceleration) extracted from the behavioral sequences (at various speeds) combined (or not) with many means of recording MPs such as electromyography ( EMG) [10], dynamic imaging techniques (e.g., X-ray Reconstruction of Moving Morphology (XROMM) and fluoromicrometry [53], and finite element analysis (FEA) [54]).

## 5. Core Concept of Neuroethological Morphology

### 5.1. Integrating Ethology and Functional Morphology

**Figure 11:**
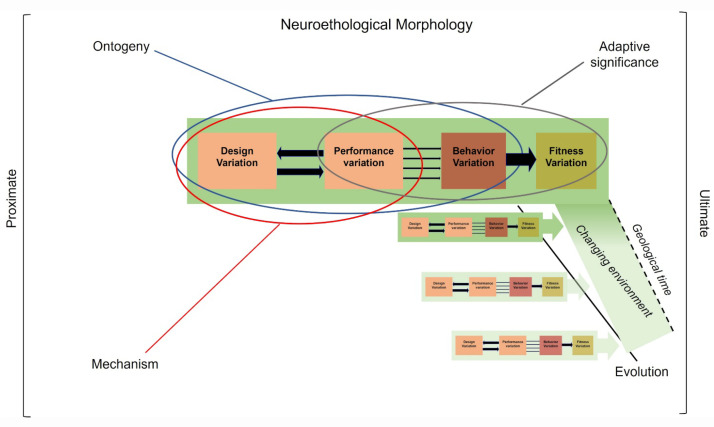
Neuroethological morphology’s heuristic approach to integrating Arnold’s paradigm [47] into Tinbergen’s four causes of behavior: ontogeny (development), mechanism, adaptive significance, and evolution. Certain links play a major role in our understanding of the causes of behaviors. The ontogeny of behavior is mainly concerned with the links between structures and performance (blue ellipse). Mechanism is primarily concerned with the links between structure, performance and behavior (red ellipse). Adaptive significance is mainly concerned with the links between performance, behavior and fitness (grey ellipse). Evolutionary pathways (black line) concern all links between structure, performances, behavior and fitness during changes in environments over geological time. Green rectangles: environments (see Figure 10).

It is salient to note that both disciplines tend to understand “how” and “why” organisms perform behaviors in response to their proximal and distal environments (Figure 11). Indeed, research in ethology and functional morphology intercept at the level of the analysis of behaviors primarily through data recorded on the sequences of animal movements and gestures. Ethologists and neuro-ethologists compare behaviors, MPs, and CPGs to understand the evolutionary mechanism(s) underlying one or several activities [55]. Functional morphologists compare “kinematics” (e.g., profiles and performances), and behavioral MPs to understand the links between “form” and “function” associated with one or more behaviors (e.g., functional trade-off) [56,57,58,59].

Integrating approaches from both disciplines can greatly contribute to the understanding of the “how” and “why” [60] in addressing questions based on the investigation of behavioral sequences [52]. The investigation of movement and MP is the main language common to both disciplines. Investigations on behavior show the need to combine data on pattern declined in kinematics, MP, and CPG with morphological and functional data (e.g., physiological and mechanical) to understand the links between structures and performance, their optimization and their limits. The integration of the ethological, morphological and functional characteristics of each animal behavior provides the holistic view to understand the action of the natural and sexual selections on the origin, development, adaptive significance, and evolution of any behavior. Such integration opens the possibility to investigate “organism–environment” interactions and to suggest hypotheses on the evolution of animal behaviors in changing environments [43].

Recently, the novel concept of mechanoethology has opened the door to the integration of ethological studies and physics by integrating physics with behavioral studies. Three major concepts play a key role in the mechanoethology: (i) energy, (ii) limits, and (iii) sensation [22]. This mechanoethology also incorporates physical approaches of organism’s responses to environmental factors summarized in the ecomechanical paradigm proposed by Higham et al. (2021) [51]. Both approaches conceptually explore responses of organisms based on physical laws applied to the properties of morphological designs and their performance. In animals, the studied responses through these concepts involve behavior determined by flexible movements, gestures, and postures.

### 5.2. Definition of Neuroethological Morphology

We propose the concept of neuroethological morphology (Figure 11) in order to build a holistic framework for understanding animal behavior. This concept integrates ethology (including behavioral ecology and neuroethology already classically associated), with functional morphology (including biomechanics and physics). This interdisciplinary concept can help understand the characteristics of all behaviors that are capable of modulating or initiating MPs to exploit respond to the environement (e.g., feeding in vertebrates [52,57,61]) and communicate [62,63]. Over the past fifty years, the parallel development of both disciplines is rooted in the study of the sequential organization of behaviors in animals. In the concept of neuroethological morphology, we propose to combine data obtained from these disciplines in order to increase our understanding of “how” animal behave, that are strongly related to questions about “organism–environment” interactions. Furthermore, combining ecological and historical constraints through the data contributes to understand “why” animals behave under particular biotic and abiotic conditions. Thus, this concept allows for the testing of evo-devo, genetic, and epigenetic hypotheses [64] to propose predictions about (i) the properties of each of the levels of the paradigm (e.g., structure, performance, behavior), and (ii) their linkages that build the “realization” of the behavioral entities (e.g., movements or gestures) across intra- and inter-individual variabilities evoked by a stimulus situation [65,66]. The tentative answers to these hypotheses open the discussion on the effect of various selective pressures (internal vs. environmental regimes) on the behaviors of organisms at any level (e.g., individual vs. group), and therefore open falsifiable predictions about the evolutionary and adaptative mechanisms of organisms under any environmental conditions (from the molecular to the behavioral level). Two examples illustrate the interest of the interdisciplinary bridges between ethology and functional morphology proposed in the concept of neuroethological morphology.

## 6. Examples of Neuroethological Morphology

### 6.1. Prey Capture in Squamates

Food/prey capture has been extensively studied in many representative squamates species [67,68], from the anatomical and mechanical properties of trophic designs (e.g., jaw and tongue), to their MPs and performance [67,68,69]. Kinematic data collected using various techniques, especially high-speed films (e.g., video and fluoroscopy techniques) and sensors (e.g., electromyography, pressure, accelerometers), mainly in experimental conditions, provide the opportunity to show the relationship between some properties of the structures, and their performance in prey capture. The holistic approach of the neuroethological morphology concept shows the bridge between ethology and functional morphology to understand “how” these tetrapods are able to exploit their food and where natural selection acts.

**Figure 12:**
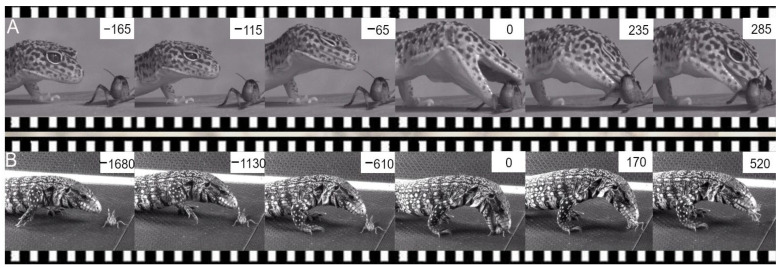
Two examples of successive images of jaw prehension in two highly different species showing the stereotyped lunge phase to catch insects. (**A**) *Eublepharis macularius*. (**B**) *Tupinambis texeguin*. Time (ms) in each frame is calculated relative to jaw contact on the prey (time = 0).

Based on the investigation of behavioral sequences in numerous species, two modes of prey capture have been determined in squamates: (i) jaw grasping (Figure 12), and (ii) lingual grasping (Supplementary Material S6) with the dorsal or the ventral side of the tongue [67,68,69,70,71,72], independent of the trophic system morphology. Each prey/food capture demonstrates invariant MPs resulting from muscular activities and kinematics of coordinated gape and hyo-lingual cycles, regardless of the morphological traits and their individual plasticity (e.g., personality). The proximal food sign stimulus acts on motor actions at two levels: (i) change in performance of the trophic system (capture mode), and (ii) modulation of trophic and post-cranial performance [71,72,73,74]. Three examples help to demonstrate the value of the neuroethological approach in understanding the evolution of prey capture in squamates. The ethological approach permits the determination of capture behavior, and the functional approach determines how structures constrain the performance of the post-cranial or trophic system.

*Iguana iguana* is an arboreal lizard mainly exploiting plant material freely available in its habitat. In adult iguanas (*Iguana iguana*), the performance of the postcranial system to capturing food is stereotyped. The lunge phase includes classic tetrapod diagonal locomotion (postcranial structures) to approach food, regardless of the substrate (e.g., flat substrate and large branches). The tongue is always protruded onto the food in a similar manner, and remains in contact with the food. In the case of wet food (e.g., juicy fruits), the lizard is able to only modulate the lingual protrusion from “*lingual prehension*” to “*tongue pinning*” (Supplementary Material S7) to ensure successful capture [67]. This arboreal lizard never changes its capture mode in jaw prehension. The postcranial system is always employed in a similar MP (e.g., gait). In contrast, the capture behavior is more complex in the scincid *Tiliqua scincoides* that forage in various habitats including forests, grasslands, and human-altered habitats to find various food resources (e.g., plants, arthropods, snails, small vertebrates). This terrestrial species may use jaw or lingual prehension for different but also similar food items [71], and lingual protrusion may be modulated differently during lingual prehension [72]. This suggests an effect of proximal sign-stimulus on the MP of the tropic system at two levels: (i) change of the jaw-tongue control, (ii) modulation of the MP of the lingual muscular system. Other lizards using lingual prehension such as *Anolis carolinensis* and *Pogona vitticeps* do not modulate their lingual movements and MP, but modulate their postcranial MP. Kinematic profiles showing coordinated jaw and lingual movements show stereotyped MP [67,68]. However, these species modulate their whole body positions to catch prey [75], in relation to proximal habitat factors (e.g., diameter, surface of the substrate). For example, *A. carolinensis* approaches the prey in a variable manner (e.g., jump capture vs. head-up capture). In the head-up capture mode, the lizard approaches the prey with its body lowered nearly parallel to the substrate, stops, and then lunges for the lingual grasp (Supplementary Material S8). The lizard secures its approach with small, continuous “postural” movements of the body and limbs.

This example emphasizes the relevance of building links between ethology and functional morphology. Ethological approach quantifies the capture behavior, particularly determining: (i) the use of the cranial and postcranial systems, and (ii) the prehension modes (lingual vs. jaw). Functional morphology investigations compare kinematic profiles and MP of body and lingual movements. Combining these two approaches in a neuroethological conceptual approach (Figure 11) allows us to show “how” different species are able to respond to proximal environmental features (e.g., food properties and substrate characteristics) by integrating the properties and links between the structure and fitness of the cranial and postcranial systems in prey capture. The integrated interdisciplinary approach provides a comprehensive understanding of the evolution of capture behavior in these tetrapods and tests the effect of proximal environment complexity on evolutionary pathways (e.g., crossing prey properties and habitat with historical constraints) of the links between the “moving morphology” and behavior.

### 6.2. Ritualized Behaviors

It is commonly accepted that a majority of the behaviors used as signals in communication are « ritualized », as stated by Lorenz (1966), who defines ritualized behavior as follows: “*…A phylogenetically adapted motor pattern which originally served the species in dealing with environmental necessities, acquires a new function, that of communication*” [76]. Ritualization is considered a key evolutionary process driving the evolution of the pattern of movements, gestures, and postures involved in animal communication (man included [77]), as notified by Morris: “*Now, it is a fundamental characteristic of the communicatory behaviour of animals that the signal patterns used are derived from non-signal sources (see, in particular, Daanje 1950, Tinbergen 1952, and Morris, 1956a. This signalisation may occur either in the phylogeny of an animal (when it is called ritualization), or in its ontogeny (when it may be termed stylization). In either case it results in the need for a fundamentally variable reaction becoming constant in form. These conflicting demands, of variability and stability, lead in many cases to a compromise: Typical intensity.*” [78]. The ritualized evolve from behaviors that can be considered originally as “cues”, and it is discussed that ritualized behaviors are one of the three pathways to explain the rise of communication and its evolution (e.g., ritualized behavior, sensory manipulation, and transformation of non-communication behavior). The interpretation of the origin of behaviors used in communication still remains unclear, but these authors suggest two stages in the emergence of ritualized behavior acting in communication: (i) coercion, and (ii) becoming a signal [79]. Display behavior is considered improbably evolved “*de novo*”. Non-communicative behaviors, which may have “preadapted” value for communication, have somehow evolved into ritualized behavior with a communication function [80]. The possible origins of ritualized behavior are numerous, and it is suggested that the MPs of ritualized behaviors derive from a wide variety of MPs of other behaviors such as (i) rhythmic behavior, (ii) displacement behavior produced by the somatic nervous system [81], (iii) ambivalent attack and flight behaviors, and (iv) intention movements are often considered at the origin of ritualized behavior [29,82,83]. To our knowledge, only a few studies demonstrate the origin of ritualized behavior on the basis of integrated neuroethological data [83,84,85,86,87,88].

Here, we suggest that the holistic approach of the neuroethological morphology concept allows us to address the question of the origin and co-evolution of ritualized MP and hyoid morphological devices in squamates. The case study of these behaviors is mainly based on (i) recorded data from photography/filming techniques associated with various sensors, and (ii) some experimental studies [89] of the throat skin and hyoid morphological properties [90,91,92].

**Figure 13:**
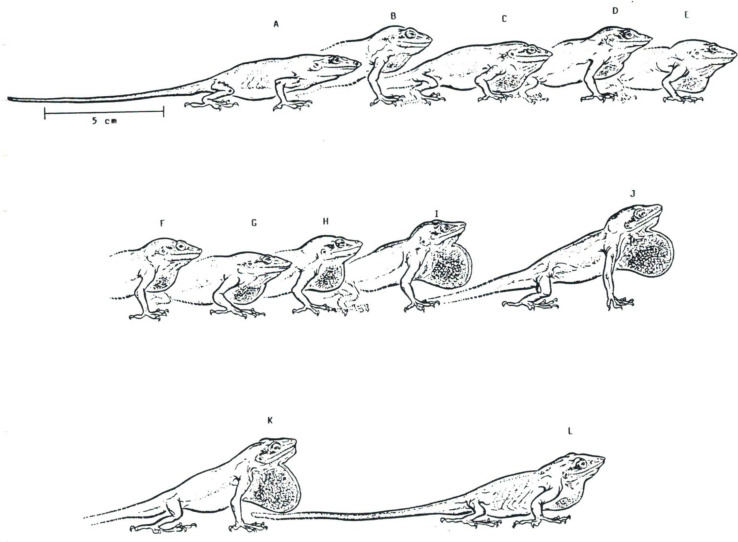
(**A**–**L**) Series of postures illustrating throat display movements (dewlap) in *Anolis carlinensis*. The time between each image is 0.01 s.

**Figure 14:**
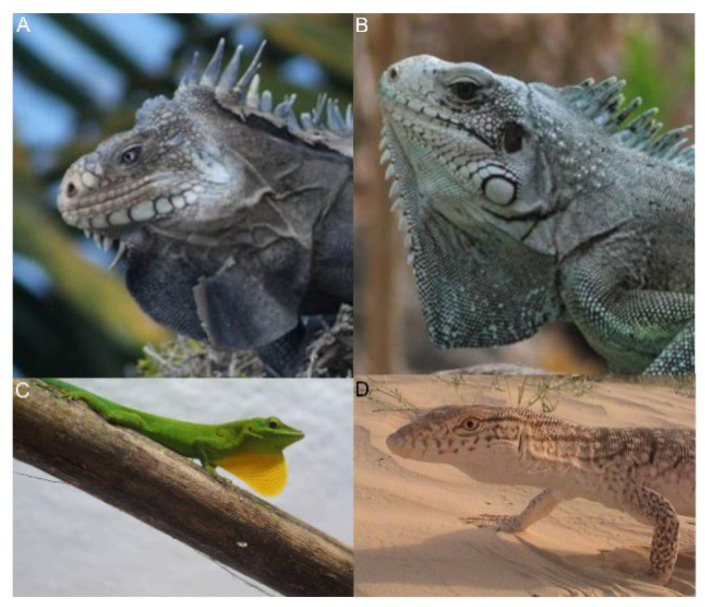
Examples of throat displays in lizards. (**A**) *Iguana delicatissima* (Archipelago of Guadeloupe). (**B**) *Iguana iguana* (Archipelago of Guadeloupe). (**C**) *Anolis marmoratus*. (**D**) *Varanus griseus*.

Throat gestures or movements are depicted in numerous species (Supplementary Material S8), as demonstrated in the pioneer work of Charles Carpenter [93]. These gestures *per se* are included (or not) in motor sequences (Figure 13), variably involving body (“push-up”) and head movements (e.g., “head-bobbing” or head-nodding”), showing the complexity of cooperation of the cranial, axial, and appendicular systems in the performance of ritualized behaviors (Figure 14).

**Figure 15:**
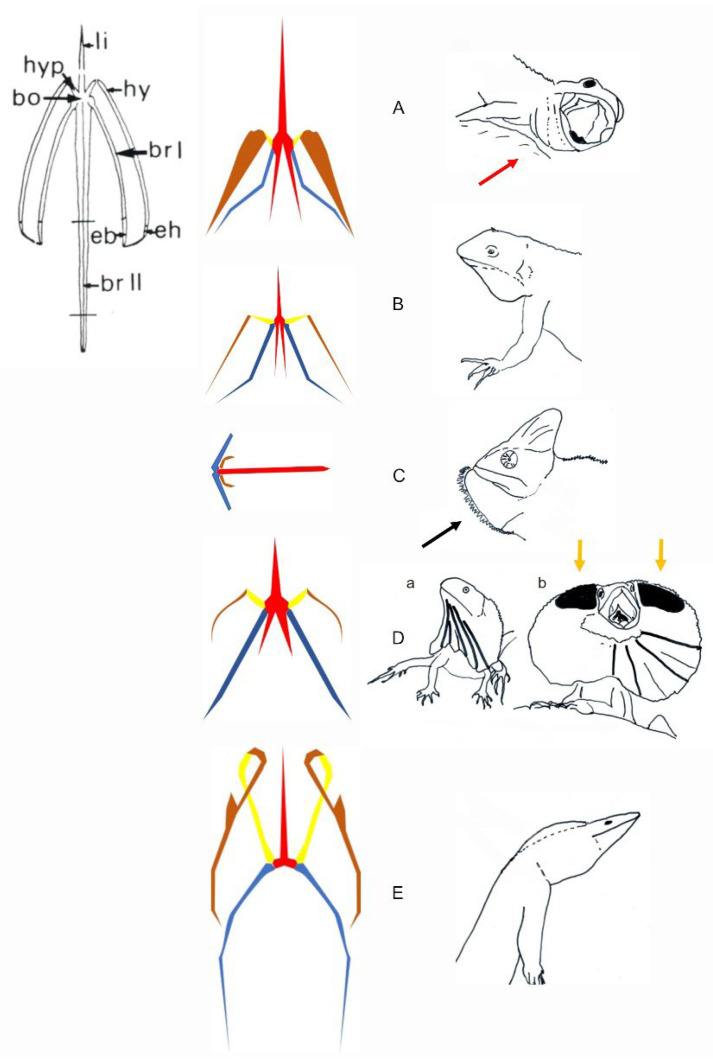
Schematic representations of the hyoid apparatus in relation to the recorded throat extension in lizards. Anatomical description of all elements of a hyoid apparatus (*Anolis carolinensis*) is provided in the upper left corner. The hyoid anatomy and display behaviors are illustrated for various lizards. (**A**) The hyoid apparatus of Lacertoidea. The display in Lacertoidea involves a very small throat extension often associated with gaping (red arrow). (**B**) Representative hyoid apparatus in Toxicofera, Pleurodonta (*Sceloporus* sp.) and Acrodonta. The display of most of the species shows a limited throat extension. (**C**) Representative hyoid apparatus in Acrodonta *Chameleo* sp. The display (illustrated in Brokesiinae) remains to be investigated functionally. The black arrow shows the potential effect of additional lingual movement on the shape of the signal. (**D**) Representative hyoid apparatus in *Chlamydosaurus kingi*. This lizard uses frill erection. a. Resting posture. b. Full frill erection. Yellow arrows indicate Grey’s cartilage (black areas) helping support dorsally the fully erected frill. (**E**) Representative hyoid in *Varanus* sp. (Toxicofera, Anguimorpha). The display in *Varanus* sp. Involves various throat extension (see Figure 16D). bo, hyoid body, br I, ceratobranchial I; br II, ceratobranchial II; eb, epibranchial; eh, epihyal; hy, ceratohyal; hyp, hypohyal; li, lingual or entoglossal process. The hyoid elements are colored following their relative movements. Red, the hyoid body, entoglossal process and ceratobranchials II in solidarity movements; yellow, hypohyal; brown, ceratohyal; blue, ceratobranchial I.

The links between morphological (e.g., cartilage, musculature), and biomechanical features of the hyoid apparatus in the display have been mainly investigated in iguanid and agamid squamates, and in some other species such as varanids [90,91,92,94,95,96,97,98,99,100,101,102,103]. The shape of the exhibited throat is highly variable within and among squamate species, as demonstrated by the enormous literature on the diversity of dewlap features (e.g., *Anolis* dewlap size and shape [98,104,105,106]). For example, the Saharan lizard *Varanus griseus* uses two types of rhythmic rapid throat movements: (i) a throat movement associated with the ventilatory cycle (and song production), and (ii) successive rhythmic rapid throat movements performed while the body remains completely inflated [101]. In *Anolis,* the throat is variably extended and pulsed in a complex sequence determined by spatiotemporal movements of the head and the body (Figure 15). In *Iguana*, the dewlap vibrates following the head bobbing (Supplementary Materials S10).

**Figure 16:**
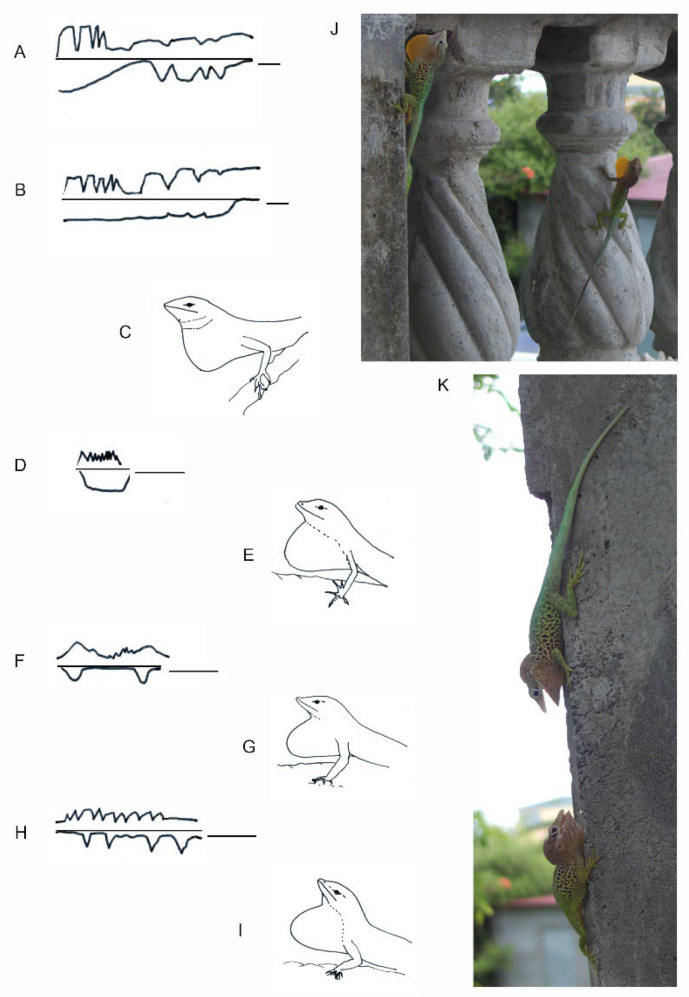
The dewlap exhibition associated with whole body movements shows different patterns and shapes in *Anolis* species. (**A**,**B**) Dewlap and headbob display showing interindividual difference in *Anolis aureus* (redrawn from [107]). (**C**) Dewlap shape in *Anolis aureus*. (**D**) Dewlap in *Anolis limifrons*. (**E**) Dewlap shape in *Anolis limifrons*. (**F**) Dewlap and headbob display in *Anolis opalinus.* (**G**) Dewlap shape in *Anolis opalinus*. (**H**) Dewlap and headbob display in *Anolis sagrei*. (**I**) Dewlap shape in *Anolis sagrei*. (**J**) Posture of maximum dewlap extension in *Anolis marmoratus* (Guadeloupe, courtesy L. Legendre, Guadeloupe). (**K**) The throat in *Anolis* can be variably extended during encounters as shown in *Anolis marmoratus* (courtesy L. Legendre, Guadeloupe), suggesting possible neuromotor control of throat extension under sensory and internal (e.g., hormonal) behavioral control. (**D**,**F**,**H**) are redrawn from [108]. The bar in (**A**,**B**): 1 s; the bar in (**D**,**F**) and (**H**): 7 s.

Neuroethological studies suggest that the MP of all ritualized throat displays in lizards is conservative. Available data (e.g., EMGs; muscular stimulations [89,90]) demonstrate that the pattern of this signal is based on the relative movements of the lateral hyoid elements, with the key role played by the muscle M. cerato (branchio) hyoideus located between the ceratohyal and the ceratobranchials I (Figure 16). Contraction of this muscle produces rotation of the hypohyals and extension of the central posterior elements, the ceratobranchials II, operating as a first-order lever in the dewlap of iguanids (e.g., *Anolis*, *Iguana*) [18,89,90,100] and in the extension *Varanus’* throat [101]. In frill erection, regardless of size and shape (e.g., *Pogona* and *Chlamydosaurus*), this muscle contraction also produces coordinated movements of the ceratobranchials I, acting directly on the skin to produce lateral expansion (*Pogona*) and frill erection (*Chlamydosaurus*).

The hyoid movements driving to all kinds of throat signal in lizards are produced by a conservative MP and CPG [83]. Throat extension and dewlap are produced by movements of the extended ceratobranchials II, and lateral expansion by the coordinated lateral movements of the ceratobranchials I.

The signal itself is affected by the morphological constraints, as shown in functional studies: (i) the 3D-shape of the hyoid (Figure 16) [83], (ii) the functional properties of the muscle systems [97,98], and (iii) the properties of the skin [92,103]. Indeed, it seems that one of the key elements that shapes the throat gesture is the morphological (e.g., scales) and biomechanical properties of the skin [92,103]. For example, skin pleats (involving three convex and two concave folds) are erected during the full frill erection in *Chlamydosaurus*, with the so-called Grey’s cartilage connecting the dorsal part of the frill to each side of the head allowing the movement of its upper part [103]. Therefore, the evolution of the shape of the signal in this agamid is the combined result of the conservative MP integrated with the properties of the skin, and the 3D-structural properties of the hyoid elements with addition of a dorsal cartilage. This additional morphological system is unique in lizards exhibiting frill erection. All other signal characteristics (e.g., color pattern) can be added (e.g., size, shape, color) to this signal by sexual selection. The origin and mechanism of throat display as part of the ritualization process remain to be investigated in squamates within the concept of neuroethological morphology. For example, the role of the tongue (when it exists) remains to be clarified in many lizard species. The neuroethological concept (Figure 11) linking performances and structure of the hyolingual apparatus permits the hypothesis that the origin of the throat display in lepidosauromorph diapids evolved only once under the process of ritualization during their evolution and change through their broad geographical distribution [109]. The co-evolution of morphological features (e.g., hyoid and skin) governs and limits the shape of the signal without affecting the other behavioral activities (e.g., feeding, drinking).

## 7. Concluding Remarks

We show the key role of the links between ethological and functional studies to support the concept of neuroethological morphology in two behaviors of lizards. On the basis of the behavioral sequences analysis, this concept integrates three disciplines that investigate the behavioral biology of animals: ethology, neuroethology, and functional morphology. This conceptual approach should be adopted for the diversity of behaviors selected by organisms in their organism–environment interactions, regardless of their biotic or abiotic environmental constraints. We suggest that this interdisciplinary approach is helpful for understanding the evolution of behavior, its origin, and adaptive significance through the investigations formalized in the Arnold’s paradigm, and permits the investigation of the evolutionary patterns and processes, such as ritualization.

## Data Availability

Data are available from the Corresponding author on request.

## Supplementary Materials

The following supporting information can be downloaded at: https://zenodo.org/record/6606197#.YpnUaCdByUl, Video S1. Preying behavior in a group of Chroicocephalus ridibundus. A lot of birds plunge independently at various heights (speed: 0.09 fps). Video S2. Sitting posture of Symphalangus syndactylus for food reaching. The animal takes his sitting posture from a bipedal erected locomotion (speed: 0.07 fps). Video S3. Sitting posture of Semnopithecus entellus for food reaching. The animal takes his sitting posture at the end of a classical quadrupedal locomotion (speed: 0.07 fps). Video S4. Semnopithecus entellus grasping a piece the food “one-shot” movement (speed: 0.09 fps). Video S5. Semnopithecus entellus uses finger rhythmic movements to screen the food before grasping one piece (speed: 0.09 fps). Video S6. Lingual prehension in Anolis carolinensis on flat substrate (speed: 0.05 fps). Video S7. Typical response of Iguana iguana facing to a wet food (fruit). The lizard always uses the tongue to contact the food, and modulates the lingual and jaw movements in the two successive attempts to be able to catch the wet fruit (Speed: 0.016 fps). Video S8. Lingual prehension of Anolis carolinensis on an artificial branch. The lizard controls the prey approach by postural changes of the body (Speed: 0.05 fps). Video S9. Throat display and head bobbing in Anolis ferreus in the Archipelago of Guadeloupe (Marie-Galante) (Speed: 0.05 fps). Video S10. Throat display and head bobbing in the alien Iguana iguana in the Archipelago of Guadeloupe (Speed: 0.09 fps).
